# Dental Scaling Decreases the Risk of Parkinson’s Disease: A Nationwide Population-Based Nested Case-Control Study

**DOI:** 10.3390/ijerph15081587

**Published:** 2018-07-26

**Authors:** Chang-Kai Chen, Jing-Yang Huang, Yung-Tsan Wu, Yu-Chao Chang

**Affiliations:** 1 School of Dentistry, Chung Shan Medical University, No. 110, Sec. 1, Jianguo N. Road, Taichung City 402, Taiwan; leokai1105@gmail.com; 2 Section of Dentistry, Zuoying Branch of Kaohsiung Armed Forces General Hospital, Kaohsiung 813, Taiwan; 3 Department of Periodontology, Tri-Service General Hospital, School of Dentistry, National Defense Medical Center, Taipei 114, Taiwan; 4 Department of Medical Research, Chung Shan Medical University Hospital, Taichung 402, Taiwan; wchinyang@gmail.com; 5 Department of Physical Medicine and Rehabilitation, Tri-Service General Hospital, School of Medicine, National Defense Medical Center, Taipei 114, Taiwan; crwu98@gmail.com; 6 Department of Dentistry, Chung Shan Medical University Hospital, Taichung 402, Taiwan

**Keywords:** Parkinson’s disease, dental scaling, periodontitis, oral health

## Abstract

The protective effect of dental scaling in Parkinson’s disease (PD) remains inconclusive. The aim of this study was to analyze the association between dental scaling and the development of PD. A retrospective nested case-control study was performed using the National Health Insurance Research Database of Taiwan. The authors identified 4765 patients with newly diagnosed PD from 2005 to 2013 and 19,060 individuals without PD by matching sex, age, and index year. In subgroup 1, with individuals aged 40–69 years, individuals without periodontal inflammatory disease (PID) showed a protective effect of dental scaling against PD development, especially for dental scaling over five consecutive years (adjusted odds ratio = 0.204, 95% CI = 0.047–0.886, *p* = 0.0399). In general, the protective effect of dental scaling showed greater benefit for individuals with PID than for those without PID, regardless of whether dental scaling was performed for five consecutive years. In subgroup 2, with patients aged ≥70 years, the discontinued (not five consecutive years) scaling showed increased risk of PD. This was the first study to show that patients without PID who underwent dental scaling over five consecutive years had a significantly lower risk of developing PD. These findings emphasize the value of early and consecutive dental scaling to prevent the development of PD.

## 1. Introduction

Parkinson’s disease (PD) is a gradually neurodegenerative disease involving deficits of the nigrostriatal pathway and characteristic motor, cognitive, and psychiatric symptoms [1]. PD onset usually occurs after 40 years of age and mainly occurs in men, with the incidence increasing with age and an annual growth rate of 7.9% [2,3]. Moreover, old-age PD onset (>70 years) is associated with greater clinical impairment [4] because rapid degeneration of the nigrostriatum and the reduced compensatory mechanism could account for faster disease progression and lead to more quantitative damage [5].

Chronic inflammatory responses such as those in periodontal problems are probably one of the etiological causes of PD [6]. The associated pathophysiology could be that periodontal disease induces host cells to generate and release pro-inflammatory cytokines such as interleukin-1 (IL-1), interleukin-6 (IL-6), tumor necrosis factor-alpha (TNF-α), and reactive oxygen species (ROS) to induce the development of PD [7]. Thus, poor periodontal status is associated with an increased risk of PD. Indeed, Chen et al. [8] revealed that patients with periodontal inflammatory disease (PID) have a greater risk of developing PD (adjusted hazard ratio = 1.431). Thus, improvements in oral hygiene may be a protective factor against PD development. Dental scaling can remove calculus and plaque and help to reduce periodontal inflammation [9]. However, there has been no direct investigation on the protective effect of dental scaling against PD development. Therefore, we conducted a nested case-control study using the National Health Insurance Research Database (NHIRD) of Taiwan to estimate the risk of PD development after dental scaling.

## 2. Material and Methods

### 2.1. Data Sources

The National Health Insurance Program was developed and managed for research purposes and provides universal and comprehensive healthcare to about 99% of Taiwanese residents [10]. The NHIRD entries from 2000 to 2013 were selected. The data used in the present study were retrieved from the information of one million randomly selected subjects in the entire NHIRD, representing about 4.5% of the entire NHIRD enrollee population [11]. There was no significant difference in age and gender between the one million randomly sampled datasets and enrollees in the NHIRD. The demographic information gathered included encrypted identification numbers, sex, dates of birth and death, diagnostic data, and procedures. The diagnostic data included the dates of dental procedures and the International Classification of Diseases, Ninth Revision, Clinical Modification (ICD-9-CM) diagnostic and procedure codes [12]. This study was approved by the Institutional Review Board (IRB) at Chung Shan Medical University (CS2-15071).

### 2.2. Study Design and Sampled Individuals

Patients who were aged ≥40 years with a new diagnosis of PD (ICD-9-CM code: 332.0) between 1 January 2005 and 31 December 2013 were enrolled [3]. Exclusion criteria were as follows: age/gender unknown and PD diagnosis before 2004. A total of 4765 individuals with PD were enlisted as the case group. In addition, 19,060 individuals without PD matched by gender, age, and index years in a 1:4 ratio were included as a control group.

Both groups were divided into two subgroups consisting of individuals with and without PID, based on the ICD-9-CM diagnostic criteria codes 523.1 (chronic gingivitis) and 523.4 (chronic periodontitis). In addition, each enlisted individual was considered to have been definitively diagnosed as having PID if the patient had at least three outpatient clinic visits for PID during a one-year study period [13]. Treatment procedures meant at least one dental scaling session in each one-year interval before the index date and were divided into three categories (no scaling; received, but not for five consecutive years; and received for five consecutive years) based on the procedure codes 91004c (full mouth scaling and oral hygiene instruction) and 91003c (localized scaling). Dental scaling is defined as supragingival scaling to remove the plaque and calculus for preventive oral health.

**Figure 1:**
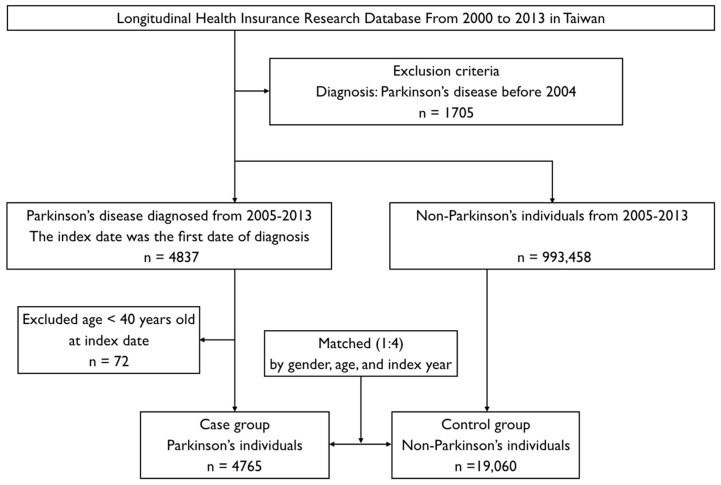
Flowchart of case-control selection of patients from the National Health Research Institute.

For both groups, we retrospectively identified the co-morbidities (including PID) and dental care utilization before the index date, as shown in Figure 1. The covariates included sex and age group (40 to 69 years, and ≥70 years). Based on the statement of urbanization issued by the National Institute of Health in Taiwan, all 365 townships from Taiwan were divided into seven clusters according to the following variables: population density (people/km^2^), the proportion of the population with educational levels of college or above, population ratio of elderly people (over 65 years old), the population ratio of people who are agricultural workers, and the number of physicians per 100,000 people. In the present study, we operationally defined townships of clusters 1–2 as level 1, clusters 3–4 as level 2, and clusters 5–7 as level 3 [14].

Data for PD-related comorbidities including PID, diabetes mellitus (DM) (ICD-9-CM code: 250.0), hypertension (ICD-9-CM codes: 401.1, 401.9, 402.10, 402.90, 404.10, 404.90, 405.1, and 405.9), hyperlipidemia (ICD-9-CM codes: 272.0–272.9), chronic kidney disease (CKD) (ICD-9-CM codes: 580, 581–589, 753, 403, 404, 250.4, 274.1, 440.1, 442.1, 447.3, 572.4, 642.1, and 646.2), depression (ICD-9-CM code: 311), stroke (ICD-9-CM codes: 433, 434, and 436), and traumatic brain injury (TBI) (ICD-9-CM codes: 800–804, 850–854, 905.0, 950.1, 950.3, 907.0, 959.01, 959.9, 310.2, and V15.52) [6,8,15,16,17,18,19] were also recorded.

### 2.3. Statistical Analysis

The chi-square test was used to compare the demographic and clinical characteristics of individuals with PD versus those without PD. Univariate and multivariate models were used to determine the odds ratio (OR) and the 95% confidence interval (CI) by conditional logistic regression models. Multivariable models were adjusted for PD-related comorbidities and urbanization level. We conducted subgroup analyses (40–69 and ≥70 years old) to account for the age-related factors identified in the development of PD in a previous study [8]. Sensitivity analysis was performed to determine the stability and accuracy of the statistical model [20]. All statistical analyses were performed using SAS version 9.3 (SAS Institute, Cary, NC, USA) and SPSS software version 22 (SPSS Inc., Chicago, IL, USA). Statistical significance was defined by a *p*-value < 0.05.

## 3. Results

The baseline demographic characteristics at the beginning of the study are shown in Table 1. Patients with PD had a higher prevalence of PID, DM, hypertension, hyperlipidemia, CKD, depression, stroke, and TBI, and showed higher urbanization levels than the control group (*p* < 0.0001).

There were 4765 PD patients and 19,060 non-PD individuals in the case and control groups, respectively (Figure 1). Table 2 shows the findings of the multivariable analysis of risk factors associated with the development of PD. In subgroup 1, among individuals aged 40–69 years and without PID, dental scaling for five consecutive years showed a protective effect against PD development in comparison with those who did not undergo dental scaling (adjusted odds ratio (aOR) = 0.204, 95% CI = 0.047–0.886, *p* = 0.0339). In contrast, among individuals with PID, dental scaling did not show a significant protective effect even among those who underwent the procedure for five consecutive years (all *p* > 0.05). In subgroup 2, among individuals aged ≥70 years, individuals without PID who did not undergo dental scaling over five consecutive years had a significantly higher risk of developing PD when compared with individuals having no dental scaling (aOR = 1.171, 95% CI = 1.026–1.336, *p* = 0.0192). Moreover, among individuals with PID, having less than five consecutive years of dental scaling was a significant risk factor for developing PD (aOR = 1.160, 95% CI = 1.008–1.336, *p* = 0.0387 for the no treatment subgroup, and aOR = 1.234, 95% CI = 1.123–1.356, *p* < 0.0001 for the subgroup without five consecutive years of dental scaling).

We wanted to confirm that dental scaling could decrease the development of PD. The results of the sensitivity analysis applied to the strategic evaluation using a logistic regression model for calculating the risk of PD after dental scaling are shown in Table 3. We applied the sensitivity analysis to assess the effect after dental scaling over one to five consecutive years before the index date in patients without PID. The association of the protective effect between dental scaling and PD remained consistent. Moreover, the protective effect tended to become more prominent with increasing consecutive years of dental scaling (aOR values for one year and five years were 0.974 and 0.204, respectively) and reached significance at three consecutive years (aOR = 0.479, 95% CI = 0.23–0.998, *p* = 0.049) to five consecutive years (aOR = 0.204, 95% CI = 0.05–0.886, *p* = 0.034).

## 4. Discussion

This was the first nationwide, population-based nested case-control study to reveal that individuals without PID who underwent dental scaling for five consecutive years had a significantly decreased risk of developing PD (aOR = 0.204) without considering comorbidities and urbanization level. Overall, our findings suggested that individuals without PID who underwent dental scaling showed a greater protective effect than those with PID, and this tendency was more predominant in the group aged 40 to 69 years than in those aged more than 70 years.

Periodontal microorganisms consist of Gram-negative bacteria, which produce the endotoxin lipopolysaccharide. The associated periodontal inflammation could also result in the breakdown of the blood-brain barrier, thereby progressively contributing to the etiology of disabling neurodegenerative disease [1,21]. PID is a chronic inflammatory condition of the tooth and is one of the most common chronic infections. This condition is associated with persistent systemic inflammation leading to elevated concentrations of C-reactive protein and other inflammatory biomarkers including IL-1, IL-6, and TNF-α [22]. These cytokines activate microglial cells that produce nitric oxide and reactive oxygen species (ROS), resulting in the death of dopaminergic neurons [1]. Therefore, systemic inflammation may represent a potential mechanism connecting oral health and neurodegenerative diseases. Thus, poor oral hygiene is a major cause of periodontal disease and has been found to be a potential risk factor for neurodegenerative diseases such as Alzheimer’s disease and PD [8,23].

Teeth cleaning is a useful and easy way to maintain optimal oral hygiene and prevent the onset of gum disease. Furthermore, a previous study confirmed that dental scaling could significantly reduce the number of oral microbial communities [24] and the risk of transient bacteremia [25]. However, no previous study has established the association between dental scaling and PD development. Our findings showed that among individuals aged 40–69 years, those who underwent dental scaling frequently (at least once a year) mostly had a relatively lower ratio of PD development, especially those individuals without PID who underwent dental scaling for five consecutive years (aOR = 0.20, *p* = 0.0339). Although individuals with PID may show a different degree of periodontal destruction, those who underwent regular dental scaling for five consecutive years showed a tendency towards an insignificant reduction in the odds of developing of PD by 28.2% (aOR = 0.718, 95% CI = 0.495–1.04, *p* = 0.0796). In general, the protective effect of dental scaling was more beneficial in individuals with PID than in those without PID, regardless of the duration of consecutive dental scaling. On the other hand, the benefits of dental scaling were not noticeable among older individuals (subgroup 2, ≥70 years old). Conversely, individuals with or without PID who did undergo dental scaling over five consecutive years and those with PID who did not undergo dental scaling at all were at high risk of developing PD (all *p* < 0.05) (Table 2).The discrepancy between subgroup 1 (40–69 years old) and subgroup 2 (≥70 years old) may indicate a unique phenotype of PD with age-related morbidity that may be related to different causes such as greater degeneration of nigrostriatum, lower compensatory repair mechanism, and more complicated comorbidities [4,5]. Further research is needed in these aspects.

We conducted a sensitivity analysis to assess the role of periodic dental scaling in PD development. We further confirmed that the aOR during the one-year to five-year follow-up period for patients aged 40–69 years without PID decreased from 0.974 to 0.204 and reached significance through three to five consecutive years of dental scaling (*p* < 0.05), relative to the no-treatment group. However, in the group of patients aged 70 years or above, individuals with or without PID mostly had a greater aOR than no treatment, regardless of dental scaling. Hence, we emphasized the value of early and at least three consecutive dental scaling sessions in preventing PD development, especially in individuals aged 40 to 69 years. In other words, it may be too late to consider the protective role of dental scaling for PD in individuals aged more than 70 years.

Our study had the following advantages: (1) we performed a NHIRD search and enlisted a large sample size in emphasizing the aOR over the five-year follow-up period; (2) the NHIRD provided continued coverage for the entire population and thus avoids selection bias; (3) the use of the NHIRD eliminates the need to minimize patients that were lost to tracing in the case-control study; (4) with regard to socio-demographic characteristics, it was easy to obtain data for a large number of geographically dispersed individuals, thereby avoiding regional discrepancies [14]; and (5) we applied a rigorous definition to identify PD patients (ICD-9-CM code: 332.0), therefore making statistical analyses more accurate and reliable.

However, our study also had some limitations: (1) we excluded individuals who had PD before tracing and ensured accuracy in patient recruitment, however, we could not differentiate between primary and secondary PD on the basis of diagnostic codes in the NHIRD [3]; (2) we did not obtain the medical records of all defined PID and PD cases as all of the NHIRD medical records were de-identified for ethical reasons; thus, clinical data such as image findings, clinical photographs, and examinations of the periodontal disease and laboratory data or treatment responses in the identified cases could not be recorded; (3) periodontal treatment in clinics, oral hygiene from caregivers, and improved education regarding good oral hygiene practices may help prevent PD by reducing inflammation [26], however, personal details on periodontal treatment were not included in the NHIRD; and finally (4), our method to extract data from the NHIRD enabled long-term follow-up periods and in the future could include additional influences such as environmental conditions, lifestyle factors (e.g., smoking), and genetic polymorphisms. Accurate risk assessment for PD in the context of PID is necessary if it is to influence healthcare planning and the national health insurance policy.

## 5. Conclusions

This was the first study to reveal that patients without PID who underwent dental scaling over five consecutive years had a significantly lower risk of developing PD. The authors emphasize the value of early and at least three consecutive dental scaling sessions to prevent the development of PD, especially in individuals aged between 40 and 69 years.

## Figures and Tables

**Table 1:** Characteristics among the Parkinson’s cases and age- and sex-matched controls at index date.

Variables	Control Group without PD *n* = 19,060	Case Group with PD *n* = 4765	*p*-Value
Sex			1.0000
Female	9624 (50.49%)	2406 (50.49%)	
Male	9436 (49.51%)	2359 (49.51%)	
Age			1.0000
40–69	5552 (29.13%)	1388 (29.13%)	
≥70	13,508 (70.87%)	3377 (70.87%)	
Urbanization			<0.0001
Level 1	10,369 (54.40%)	2440 (51.21%)	
Level 2	5642 (29.60%)	1256 (26.36%)	
Level 3	3049 (16.00%)	1069 (22.43%)	
Comorbidities			
Periodontal inflammatory disease	8554 (44.88%)	2322 (48.73%)	<0.0001
Diabetes mellitus	6925 (36.33%)	2146 (45.04%)	<0.0001
Hypertension	13,151 (69.00%)	3784 (79.41%)	<0.0001
Hyperlipidemia	9066 (47.57%)	2601 (54.59%)	<0.0001
Chronic kidney disease	4996 (26.21%)	1658 (34.80%)	<0.0001
Depression	646 (3.39%)	447 (9.38%)	<0.0001
Stroke	3217 (16.88%)	1669 (35.03%)	<0.0001
Traumatic brain injury	5678 (29.79%)	2598 (54.52%)	<0.0001
Dental scaling *			<0.0001
No treatment	9773 (51.27%)	2282 (47.89%)	
Not 5 consecutive years	8615 (45.2%)	2336 (49.02%)	
For 5 consecutive years	672 (3.53%)	147 (3.08%)	

* The utilization of dental scaling was identified within the 5-year period prior to index date. PD: Parkinson’s disease.

**Table 2:** Odds ratio of Parkinson’s disease for dental scaling * and periodontal inflammatory study among the cases and controls, with subgroup analysis.

Variables	Individuals	Multivariable Analysis		
		aOR	95% CI	*p*-Value
Subgroup 1: 40–69 years old				
No treatment without PID	2336	Reference	-	-
Not 5 consecutive years without PID	1001	0.943	0.770–1.155	0.5698
For 5 consecutive years without PID	35	0.204	0.047–0.886	0.0339
No treatment with PID	515	1.030	0.801–1.324	0.8159
Not 5 consecutive years with PID	2781	1.056	0.908–1.228	0.4763
For 5 consecutive years with PID	272	0.718	0.495–1.040	0.0796
Subgroup 2: ≥70 years old				
No treatment without PID	7645	Reference	-	-
Not 5 consecutive years without PID	1866	1.171	1.026–1.336	0.0192
For 5 consecutive years without PID	66	1.132	0.604–2.124	0.6986
No treatment with PID	1559	1.160	1.008–1.336	0.0387
Not 5 consecutive years with PID	5303	1.234	1.123–1.356	<0.0001
For 5 consecutive years with PID	446	1.133	0.885–1.452	0.3217

* The utilization of dental scaling was identified within 5 years before the index date. The multivariable analyses were adjusted for PID, diabetes mellitus, hypertension, hyperlipidemia, chronic kidney disease, depression, stroke, traumatic brain injury, urbanization level. aOR: Adjusted odds ratio; CI: Confidence interval; PID: Periodontal inflammatory disease.

**Table 3:** Sensitivity analysis of case-control model for Parkinson’s disease.

Annual Dental Scaling without PID	aOR	95% CI	*p*-Value
Within 1 year before index date	0.974	0.731–1.297	0.856
2 consecutive years before index date	0.742	0.464–1.187	0.213
3 consecutive years before index date	0.479	0.23–0.998	0.049
4 consecutive years before index date	0.233	0.07–0.774	0.017
5 consecutive years before index date	0.204	0.05–0.886	0.034

PID: Periodontal inflammatory disease; aOR: Adjust odds ratio; CI: Confidence interval.
